# Supplementary material for: Electric-field-assisted proton coupling enhanced oxygen evolution reaction
Source: Nat Commun. 2024 Apr 18;15:3354. doi: 10.1038/s41467-024-47568-y (PMC11026508; doi:10.1038/s41467-024-47568-y)
Supplement: Supplementary file 1 — Supplementary information [file 41467_2024_47568_MOESM1_ESM.pdf]

Supplementary Materials for

Electric-field-assisted Proton Coupling Enhanced Oxygen Evolution Reaction

Xuelei Pan[1,2], Mengyu Yan[1]*, Qian Liu[3], Xunbiao Zhou[1], Xiaobin Liao[1], Congli Sun[1], Jiexin Zhu[1], Callum McAleese[4], Pierre Couture[4], Matthew K Sharpe[4], Richard Smith[4], Nianhua Peng[4], Jonathan England[4], Shik Chi Edman Tsang[2]*, Yunlong Zhao[5,6]*, Liqiang Mai[1]*

[1]State Key Laboratory of Advanced Technology for Materials Synthesis and Processing, International School of Materials Science and Engineering, Wuhan University of Technology, Wuhan, 430070, P.R. China

[2]Wolfson Catalysis Centre, Department of Chemistry, University of Oxford, OX1 3QR, UK

[3]School of Materials Science and Engineering, Zhejiang University, Hangzhou, 310027, P. R. China

[4]UK National Ion Beam Centre, University of Surrey, Guildford, Surrey, GU2 7XH, UK

[5]Dyson School of Design Engineering, Imperial College London, London, SW7 2BX, UK

[6]National Physical Laboratory, Teddington, Middlesex, TW11 0LW, UK

*Corresponding author. Email: ymy@whut.edu.cn (M.Y.); edman.tsang@chem.ox.ac.uk (S.C.E.T.); yunlong.zhao@imperial.ac.uk (Y.Z.); mlq518@whut.edu.cn (L.M.)

Supplementary Methods

Fitting of Raman spectra

The Raman spectra are fitted by LabSpec 6 software, where the baseline is subtracted by polynomial fitting and the peaks are fitted by Gauss-Lorentz line shape[1].

For Gauss line shape, the function is described as $G\ (v - v_0) = Ae^{\frac{-(v-v_0)}{2(\frac{\omega}{2})^2}}$. For Lorentz line shape, the function is described as $L(v - v_0) = \frac{\omega/2\pi}{(v-v_0)^2+(\frac{\omega}{2})^2}$. The Gauss-Lorentz line shape is described as $V(v - v_0) = \int G\ (v_0' - v_0)\ L(v - v_0')\ dv_0'$. In the equations, A is preexponential factor, v is Raman shift, ω is the full width at half maximum (FWHM), and v_0 is the position of the peak.

Current density calculation of single nanowire electrode

To calculate the current density of a single MnO_2 nanowire electrode in electrochemical measurement, the geometric area of the nanowire was calculated. Here the nanowire was simplified as an isodiametric cylinder model and the exposed surface area is defined as the surface area of the electrode, $S = \pi dL$, where S is the exposed area of the nanowire, d is the diameter of the nanowire and L is the length of the exposed area of the nanowire.

Sample preparation for the characterisation of MnO_2 nanowire after conditioning

To prepare the sample after conditioning, electrochemical measurements are performed with a typical three-electrode configuration, where an indium tin oxide (ITO) glass was used as the working electrode, graphite used as the counter electrode and Hg/HgO electrode was used as reference electrode. When preparing the catalyst ink, 5 mg MnO_2 nanowire powder was dropped to 1 mL ethanol and following the ultrasonic treatment. The obtained ink was dropping cast onto the ITO glass (20 mm × 25 mm, 4-6 ohm/sq) and heated at 50 °C until forming a uniform film. After test, the ITO glass was washed with deionised water and then put in ethanol and to perform following the ultrasonic treatment. The powder was obtained after drying at 70 °C.

X-ray absorption fine spectroscopy

The *in situ* XAS was tested in a fluorescence model by a homemade electrochemical cell at the beamline BL11B of Shanghai Synchrotron Radiation Facility (SSRF). The standard data analysis was performed by ATHENA and ARTEMIS modules implemented in the IFEFFIT software package[2]. The X-ray absorption near edge structure (XANES) and the extended X-ray absorption fine structure (EXAFS) spectra were processed by the software packages Athena 0.9.26. To highlight the high-energy oscillations, the EXAFS signal $\chi(k)$ was weighted by k^3 and then Fourier transformed in a range of R from 0 to 6.0 Å. The XAFS fitting models were calculated with the ab initio code FEFF 6 including scattering amplitudes, phase shifts, and photoelectron mean free paths for all paths.

DFT Theoretical calculations

The density functional theory calculations with Hubbard model (DFT+U) [3,4] were performed via the

program package DMol^3 (ref. [5,6]) in the Materials Studio (Accelrys Inc). The generalised gradient approximation (GGA) and the Perdew-Burke-Ernzerhof (PBE) exchange-correlation functional[7] was adopted. The effective Hubbard U value of 4.5 eV was applied to describe Mn(3*d*) electrons better[8]. Orbital cutoff was set to 4.7 Å and thermal smearing with a width of 0.01 Ha was applied to the orbital occupation to speed up convergence. Grimme method[9] was used to describe the Van der Waals effect. The Monkhorst-Pack[10] k-point sampling of 6×3×1 mesh was used to optimise the (010) slab and calculate the electronic properties. The convergence criteria were set to: (1) an energy tolerance of 10^{-5} Ha/atom; (2) a force tolerance of 0.002 Ha/Å; (3) a max displacement tolerance of 0.005 Å; (4) a self-consistent field (SCF) tolerance of 10^{-5} Ha/atom.

The free-energy diagram of the OER is evaluated by the method of Nørskov et al. [11]. The change of Gibbs free energy for each step was calculated as the following equation:

$$\Delta G = \Delta E + \Delta ZPE - T\Delta S + \Delta G_U + \Delta G_{\text{pH}} + \Delta G_{\text{field}},$$

where ΔE, ΔZPE and ΔS are the difference in DFT-calculated total energy difference between the reactant and the product, contributions of the zero-point energy to the free-energy change and the change in entropy between the products and reactants, respectively. T is the temperature and is taken as 298.15 K. Zero-point energy correction was performed by frequency calculations, $ZPE = \frac{1}{2}hv$, where h is Planck's constant and v is the frequency. For the calculation of surface (*) and surface adsorbed species (*OH), we denote the *ZPE* of the surface as 0. When calculating the energy of adsorbed species, the atoms of surface are fixed and only the vibration of the adsorbed species is calculated. The zero-point energy corrections and entropic contributions to the free energies can be found in Table 1. $\Delta G_U = eU$, where U is the electrode potential with respect to the standard hydrogen electrode and e is the transferred charge. ΔG_{pH} is the correction H^+ free energy by the concentration, which can be calculated through $\Delta G_{pH} = k_B T \times \ln 10 \times$ pH, where k_B is the Boltzmann constant and pH = 0 is assumed in the acidic medium in this work. ΔG_{field} is the free-energy correction resulting from the electrochemical double layer, which is neglected in the present study.

Supplementary Table 1. Zero-point energy corrections and entropic contributions to free energies. Entropies of gas-phase values are from handbook[12]. The entropy of surface and adsorbed species is ignored. The ZPEs for the gas molecules the adsorbed species are taken from DFT calculations (frequency calculations).

	S (J K^{-1} mol^{-1})	ZPE (eV)
O_2(g)	205.147	0.095
H_2O(g) (0.035 bar)	216.819	0.56
H_2(g)	130.680	0.27
*	0	0
*OH	0	0.372
*OOH	0	0.445
*O	0	0.074
O+OH	0	0.450
*OH+*OH	0	0.754

Supplementary Notes

Structural characterisation

The cation-accommodated α-MnO_2 nanowires were synthesised by a standard hydrothermal method using $KMnO_4$ and NH_4F. The X-ray diffraction (XRD) pattern of as-prepared nanowires can be indexed perfectly to the tetragonal α-MnO_2 (JCPDS No. 44-0141, Supplementary Fig. 1a. In the following text, we use α-MnO_2 for short as cation-accommodated α-MnO_2.). Raman bands with three main contributions at 183 cm^{-1}, 580 cm^{-1}, and 635 cm^{-1} indicate the typical α-MnO_2 structure (Supplementary Fig. 1b). The XPS doublet Mn 2*p* peaks show that Mn $2p_{3/2}$ and $2p_{1/2}$ locate at 642.0 and 653.8 eV, indicating the +4 valance of Mn (Supplementary Fig. 1c). The morphology and atomic structure were characterised using field emission scanning electron microscopy (FESEM) and scanning transmission electron microscopy (STEM) (Supplementary Fig. 2). The α-MnO_2 nanowires prepared in this work are monocrystalline, growing in the [001] direction with a diameter of around 40 nm (Supplementary Fig. 2b,c).

Raman spectra of MnO_2 nanowires

Raman spectra for the obtained sample are shown in Supplementary Fig. 1b. The Raman modes of α-MnO_2 contribute from Mn-O vibrations and 4 Mn and 8 O atoms are lying on an 8h site. Hence, the optical modes can be calculated as, $\Gamma_{\text{Mn-O}} = 6A_g+6B_g+3E_g+2A_u+3B_u+5E_u$. According to factor group analysis, the A_g, B_g and E_g modes are Raman active[13]. Here, we observed four apparent vibrations band features for α-MnO_2 nanowire. The sharp peak located at 183 cm^{-1} (v_0) can be attributed to the translational motion of [MnO_6] octahedral and the peak at 385 cm^{-1} is the Mn-O bending vibration. Another two main bands at 579 and 632 cm^{-1} (v_1 and v_2) are assigned to the A_g vibration bands contributing to the symmetric Mn-O vibrations. In details, v_1 and v_2 are assigned to the displacement of the oxygen atoms relative to the manganese atoms along the octahedral chains and the Mn-O-Mn vibrations perpendicular to the direction of the [MnO_6] octahedral double chains, respectively. The weak peak at 328 cm^{-1} is assigned to the Mn-O lattice vibrations in [MnO_6] octahedral.

Structural characteristics of α-MnO_2

The crystal structure of α-MnO_2 is a typical 2×2 octahedral molecular sieve (OMS) tunnel framework. The tunnels are formed by joining [MnO_6] octahedra in corner-to-corner and edge-to-edge [14], forming two kinds of bridging oxygen sites (mono-μ-oxo and di-μ-oxo). Similar to the core in Mn_4CaO_x in PSII, the oxygen-bridged manganese chain is supposed to a structural analogy to the Mn^{IV}-O-Mn^{III}-H_xO motifs. The tunnels accommodate cations, such as K^+ and H_3O^+, to stabilise the large tunnel structures and balance the negative charges, which decisively contributes to the mixed valence states of Mn and high nucleophilicity of μ-oxo[15,16]. These characteristics improve the affinity of protons into the α-MnO_2 tunnels, facilitating charge transfer, and thus allow α-MnO_2 to exhibit better catalytic activity compared to other manganese oxides[17,18]. Nevertheless, the catalytic performance of α-MnO_2 is poor compared to that of the Mn_4CaO_x core in PS II. This problem can be mechanistically attributed to the imbalanced coupling of protons and electrons or the strong oxygen affinity of Mn. It is acknowledged that the intrinsic catalytic activity is related to the 3*d* electrons of manganese, i.e. Mn sites with low occupancy of e_g orbital acts as efficient adsorption sites of oxygen intermediates,

whereas strong adsorption of intermediates actually causes a high thermodynamics barrier during oxygen evolving[19]. The OER of MnO_2 in alkaline condition includes a pre-equilibrium proton-coupled electron transfer (PCET)[20,21] pathway, i.e. the deprotonation of surface terminal oxygen[22]. However, the inherent strong nucleophilicity of μ-oxo limits the redox transition and the activity of adjacent active sites are reduced. Then the disproportionation of Mn^{3+}, the competitive process of PCET, is dominant and may induce the structural degradation and reduction in redox reaction rate[16].

The OER performance evolution of α-MnO_2 nanowire

The electrocatalytic oxygen evolution reaction performance of MnO_2 nanowire was measured in 1 M KOH with a three-electrode configuration. The performance of aggregation catalyst film is measured by linear sweep voltammetry, The overpotential to reach a current density of 10 mA/cm^2 is 490 mV and Tafel slope is 95 mV/dec (Supplementary Fig. 3), which is consistent with the previous report[17]. Herein, MnO_2 nanowires catalyst show a moderate Tafel slope lower than 120 mV/dec, which suggests that the adsorbed species remain dominant in OER process[23].

The DFT calculation results and OER mechanism

As the analogue of the oxygen evolving complex in PSII, the OER pathway on (010) surface of α-MnO_2 is proposed as conventional adsorbate evolution mechanism[17,24-26]. It is worth noting that the active site is found to consist of two Mn atoms (only for adsorption of *OOH) on the pristine MnO_2 surface, according to the calculated structure[25,27]. In this proposed pathway, the first step is concerted PCET process which contains a deprotonation of terminal oxygen and oxidation of Mn sites from the resting state. It has been demonstrated that structural phase transitions in spinel and layered phase manganese oxide are due to proton incorporated disproportionation reactions during OER[15,28]. It is rational that we can hypothesise that the protons released during the OER process in α-MnO_2 can contribute to redox transition and regulate the oxygen evolution.

The DFT calculation is performed to reveal the proton adsorption sites induced structural distortion and energy profile. Some literature has done systematic work on the structure of α-MnO_2 and proton intercalation in the manganese oxide[28-30]. The previous work uncovered that in ramsdellite structure, protons show a preference to the pyramidal oxygen site compared to the planar oxygen sites, corresponding to the di-μ-oxo sites with high accessibility of protons. Meanwhile, in the tunnel structure of manganese oxide, the protons can only transport by hopping between oxygen sites in the tunnel structures. The above conclusion is also in point in this work for α-MnO_2. In this work, we considered the difference of proton adsorption on di-μ-oxo and mono-μ-oxo sites, and found that the protons adsorption on mono-μ-oxo sites (sites 3 in Supplementary Fig. 6) is an energy unfavourable situations. Hence, we only took the di-μ-oxo sites into account and calculated the energy diagram of different protons adsorbed in tunnels and surface.

In this work, we proposed a conventional OER mechanism for MnO_2 in alkaline environment. Although the former literature has suggested dual sites coupling in lattice oxygen mechanism, involving the coupling of two adjacent *OH/*O, the calculation results show an overpotential of ~0.8 V through this pathway. Hence, a convention mechanism is rational for α-MnO_2, as follows:

$$* + OH^- - e^- \rightarrow *OH \quad (1) \quad \Delta G_1$$

$$*OH + OH^- - e^- \rightarrow *O + H_2O \quad (2) \quad \Delta G_2$$

$*O + OH^- - e^- \rightarrow *OOH$ (3) ΔG_3

$*OOH + OH^- - e^- \rightarrow * + O_2 + H_2O$ (4) ΔG_4

We further investigate the taches between the proton transfer process in MnO_2 and the deprotonation process on MnO_2 surface (OER). In the anodic process, the intramolecular proton transfer proceeds firstly, in which the lattice protons are extracted through transferring between adjacent lattice oxygen atoms in MnO_2. With the potential scanned higher, the adsorption of hydroxyl gradually dominates the nonfaradaic process which introduces the following proton-incorporated process. Hence, these two proton processes are continuous, and the former is expected to adjust the energy profile for OER. The pristine (010) facet of MnO_2 shows a moderate overpotential of 0.56 V and the potential determining step (PDS) is the deprotonation and dissociation step of *OOH (step 4) (Supplementary Fig. 15). Different from the common adsorption states on transition metal compounds, *OOH is adsorbed by two Mn sites according to the calculated results. When the di-μ-oxo adsorbed protons in tunnel structure, PDS may turn into the deprotonation of *OH (step 2) and the overpotential decreases to 0.44 V. Taking the structures with different protons configurations into account, we found three common laws for the OER process: First, the PDS of all the structure configurations is principally step 3 and step 4, although ΔG_3 is dependent on the proton configuration; Second, the free energy change of deprotonation steps (step 2 and step 4) is hardly changed by protons configuration. Third, the adjacent oxygen sites without adsorbed protons will decisively change two sites bonding of *OOH to single bonding and result in a higher free energy change (ΔG_3). Furthermore, we found that when protons are adsorbed to the surface di-μ-oxo sites, one part of the bridging oxygen bond will be broken and reform the disordered mono-μ-oxo. We also noticed that the proton configuration adjustment cannot bypass the scaling relationship between *OH and *OOH, although the *OOH intermediates are tuned (Supplementary Fig. 16). According to the results above, the energy profile of the OER process is dominated by the proton configuration and maintaining a circulation of lattice and surface protons will contribute to moderate adsorption energy OER thermodynamics.

The discussion of proton-electron mechanism of MnO_2 in alkaline media.

Supplementary Fig. 23a illustrates the extraction process of protons from the lattice, where they hop on adjacent oxygen sites. Supplementary Fig. 23b depicts the adsorption process of H^+, where H_2O dissociates into hydroxyl and adsorbed protons. These protons then couple with the oxygen in the lattice (μ-oxo). Furthermore, owing to the rotation and vibration of OH^- within the lattice, the O-H bonds are easily broken, allowing protons to jump to adjacent O sites or couple with hydroxyl.

Supplementary Figures

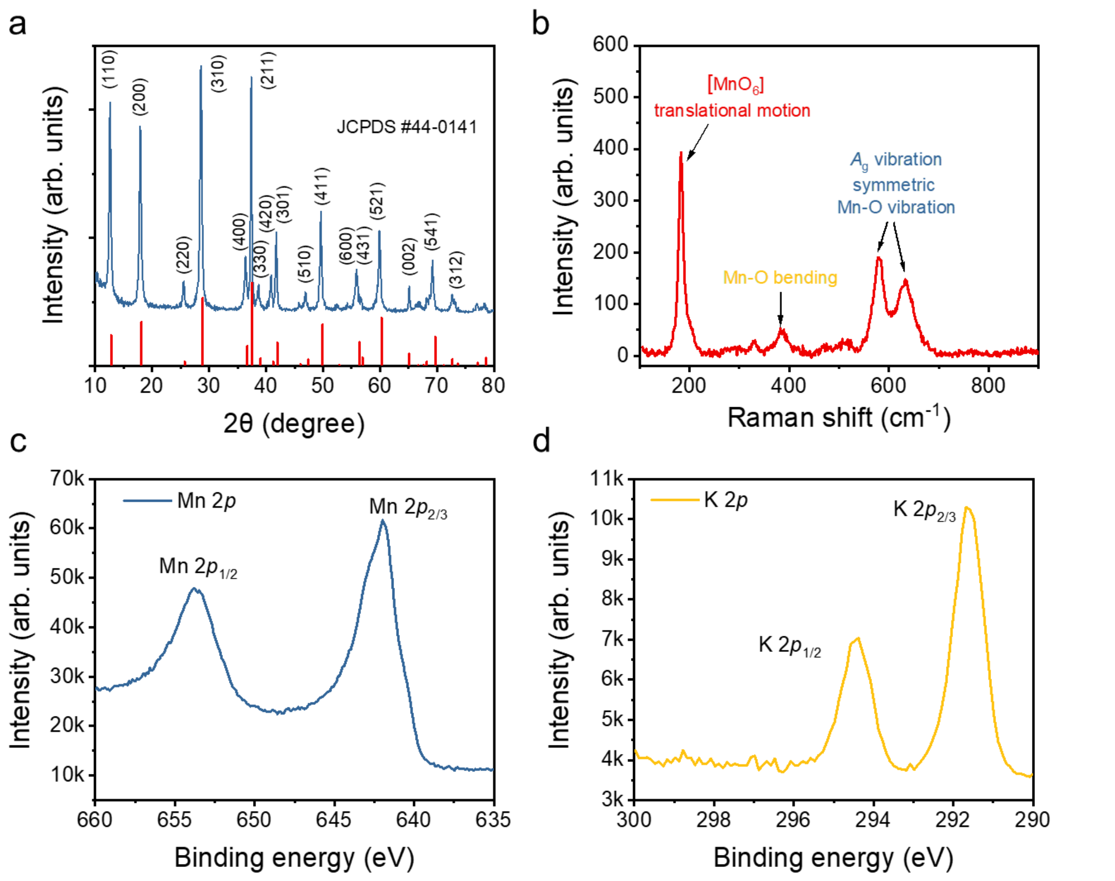

Supplementary Figure 1. Phase and chemical composition characterisation of as-prepared α-MnO_2 nanowires. (a) XRD pattern of as-prepared nanowires and corresponding JCPDS card of α-MnO_2 (No. 44-0141). (b) Raman spectrum of as-prepared α-MnO_2. Raman bands with four main contributions at 183, 385, 580, and 635 cm^{-1} are attributed to the Mn-O lattice vibrations within the [MnO_6] octahedral frameworks, featuring of the typical α-MnO_2 structure. (c-d) High-resolution XPS spectra of Mn 2*p* (c) and K 2*p* (d).

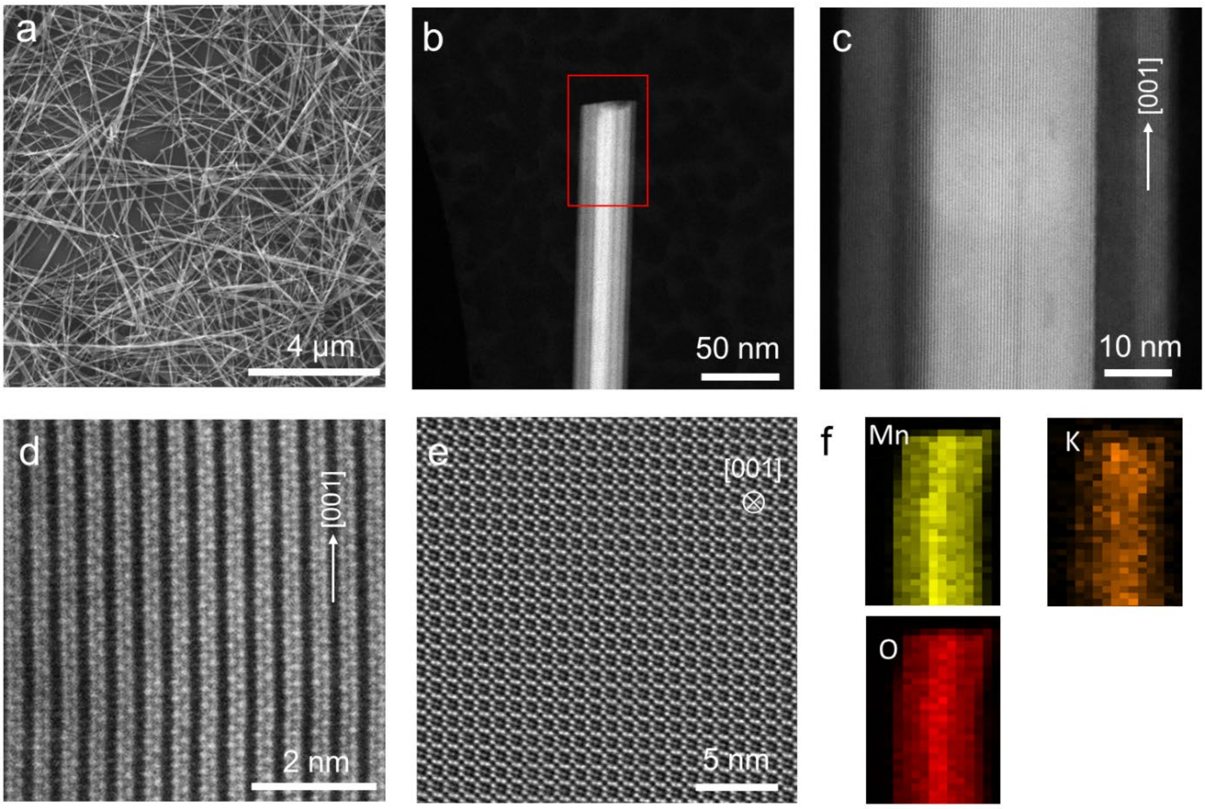

Supplementary Figure 2. Morphology and atomic structure characterisation of as-prepared α-MnO_2 nanowires. (a) FESEM image of as-prepared α-MnO_2 nanowires. (b,c) STEM image of the α-MnO_2 nanowire and corresponding elemental mapping in the red line box. (d) High-resolution STEM image of as-prepared α-MnO_2 nanowire, showing the growing direction [001]. (e) The HAADF images vertical to the directions of [001]. (f) EDS mapping of Mn, K and O, corresponding to the red box in figure b.

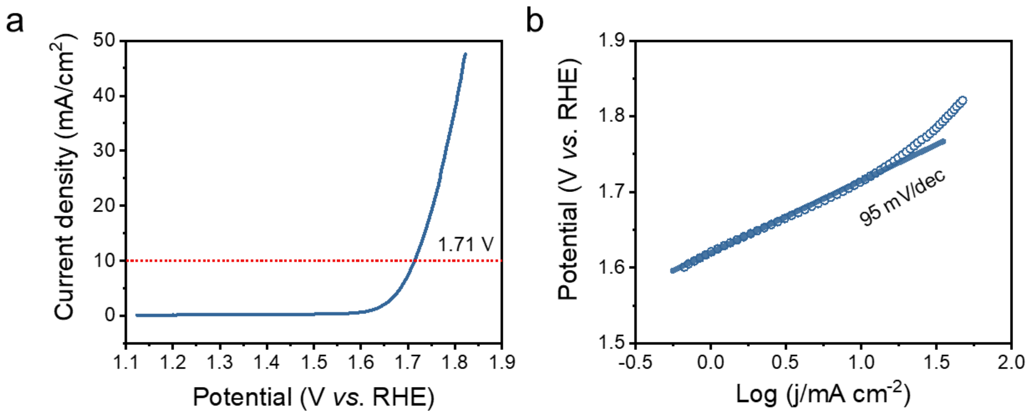

Supplementary Figure 3. Electrochemical measurement of α-MnO_2 nanowire thin film for OER. (a) Polarisation curve and (b) the corresponding Tafel plot of MnO_2 nanowire film electrode. The measurement was carried by a glassy carbon electrode (3 mm) and catalyst ink prepared by dispersing 5 mg MnO_2 and 0.05 mL Nafion solutions, 0.20 mL H_2O in 0.75 mL isopropanol. The typical mass loading of catalyst is 2.5 mg/cm^2.

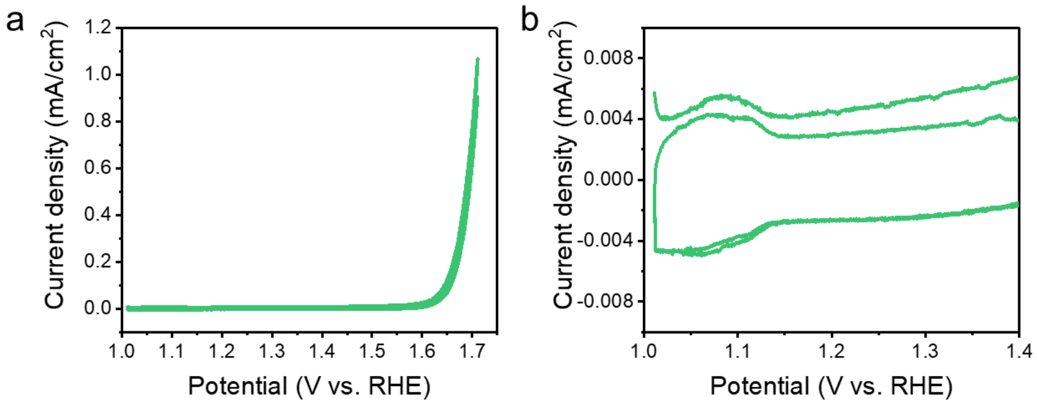

Supplementary Figure 4. The CV curves recorded during *in situ* Raman spectra measurement corresponding to the results in Fig. 1. (a) The CV curves of two cycles from 1.0 to 1.7 V vs. RHE (scan rate 0.5 mV/s). (b) the magnified CV curves from 1.0 to 1.4 V.

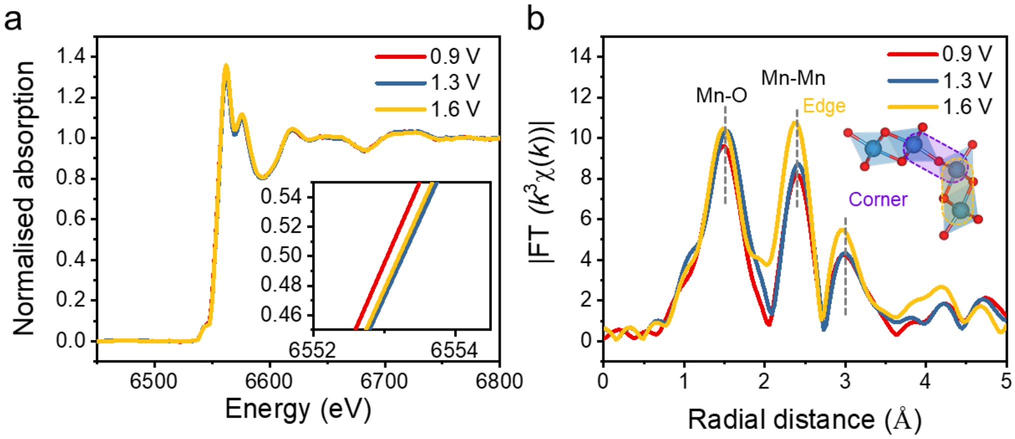

Supplementary Figure 5. The *in situ* XAS results of MnO_2 in OER process. (a) The rough Mn K-edge XANES spectra of MnO_2. Inset: the magnified part of XANES. (b) The corresponding Fourier transformations of Mn K-edge EXAFS spectra using k^3 weighting. Three peaks are depicted as Mn-O bonds, edge-shared Mn-Mn and corner-shared Mn-Mn shells.

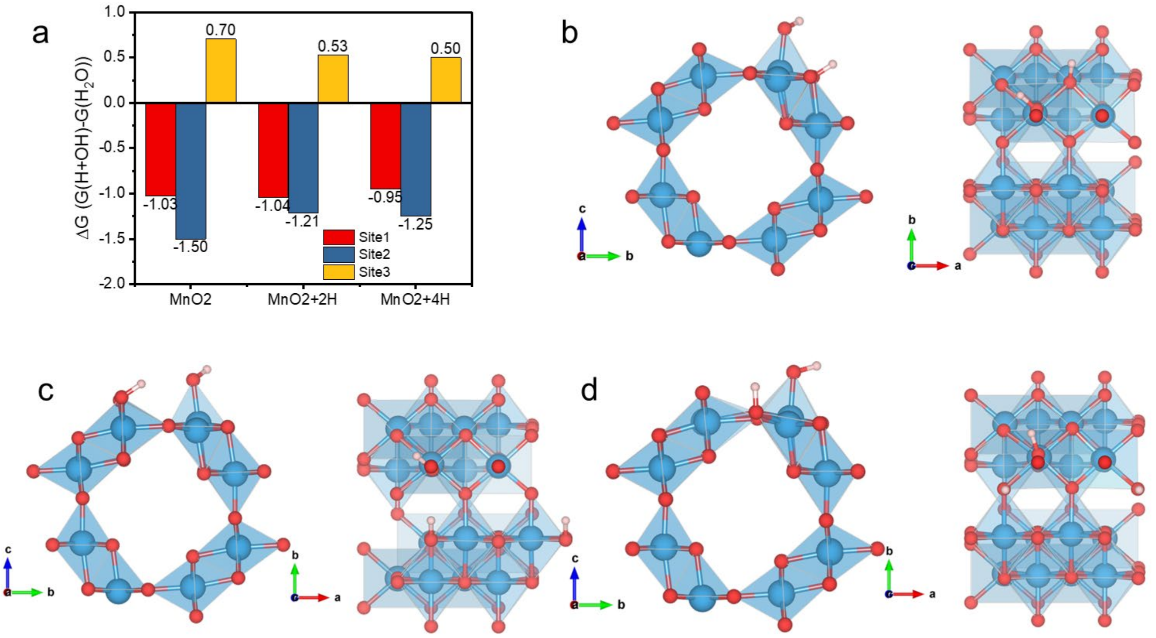

Supplementary Figure 6. Three situations of H_2O decomposed on MnO_2 (010) surface. (a) The plot of free energy changes ($G_H + G_{OH} - G_{H2O}$) of H_2O decomposition (the unit is eV). Site 1, 2 and 3 are shown in (b), (c) and (d). Here the hydroxyl is supposed to adsorbed on Mn sites and the protons are supposed to adsorbed on three possible sites. (b) shows the proton adsorbed on the adjacent di-μ-oxo site, (c) shows the proton adsorbed on the opposite protruding oxygen site, and (d) shows the shows the proton adsorbed on the adjacent di-μ-oxo site (the middle oxygen site). The free energy plot shows that the site 3 is not spontaneous, while site 2 is the most thermodynamic favourable. And we also calculated different tunnel structures with adsorbed protons, which shows that more tunnel protons will slightly increase the delta free energy. These results confirm that when tunnel protons are extracted, surface protons may increase, and the surface structure will change accordingly.

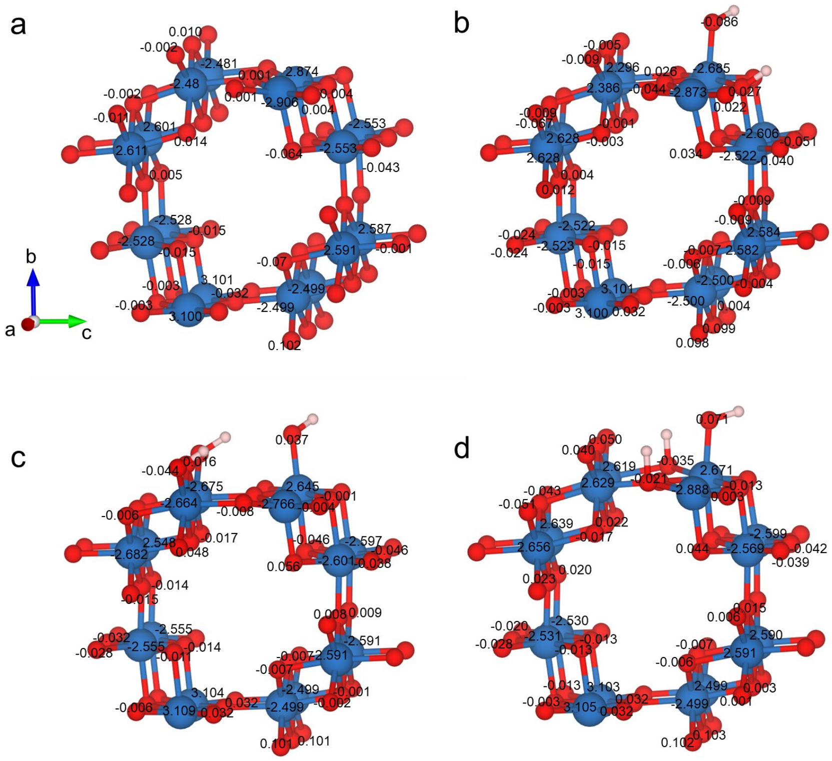

Supplementary Figure 7. The structure model of MnO_2 without and with surface protons. The labels are magnetic moment (μB). Blue, red, and pink spheres represent Mn, O and H atoms, respectively. (a) The structure model of MnO_2 without surface protons. (b-d) The structure model of MnO_2 with surface protons adsorbed on sites 1, 2 and 3, respectively. These three modes are related to Supplementary Fig. 6.

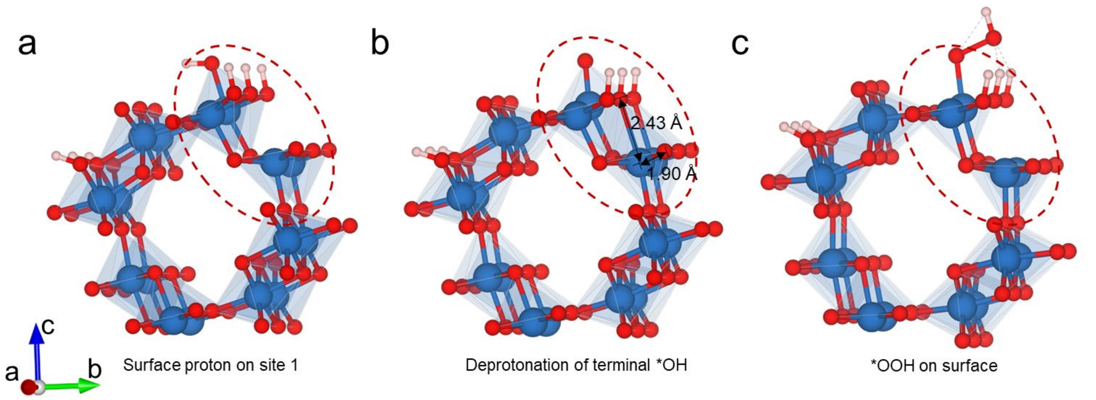

Supplementary Figure 8. The structure model of MnO_2 with surface protons on different site 1. Blue, red, and pink spheres represent Mn, O and H atoms, respectively. b and c are the following steps after a in the OER process. a represents the adsorption state of *OH, b is deprotonation of *OH and c is the formation of *OOH.

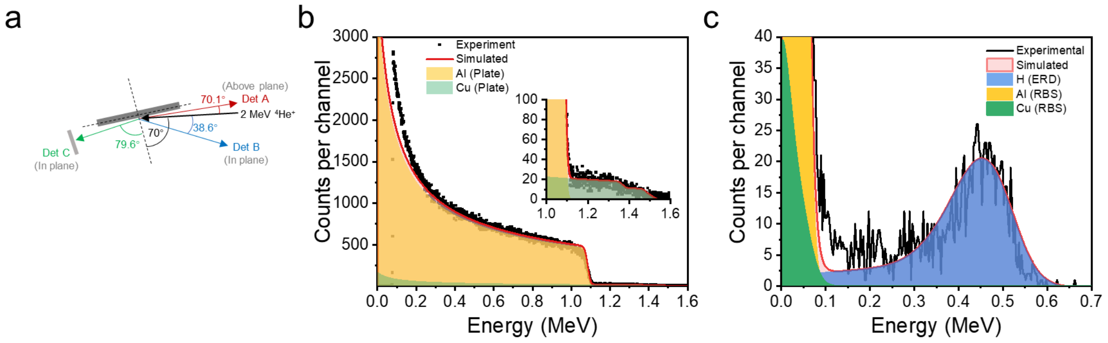

Supplementary Figure 9. Experimental and fitted non-corrected RBS (IBM geometry) and ERD spectra. (a) The schematic illustration of the setup for simultaneous ERD and RBS measurements. RBS, (b) and ERD, (c) spectra of the Al sample plate surface used to correct spectra. The inset of (b) shows the Cu RBS signal visible above the Al edge.

Supplementary Table 2. The concentrations of H, Mn, O and K atoms in the three samples obtained using ERD and RBS, adjusted for hitting the sample plate. The experimental conditions allow the concentrations to be determined down to ~8500 TFU (where a thin film unit, TFU, is 10^{15} atoms / cm^2). Uncertainties in the values have not been rigorously calculated. Counting statistics were ~ 1% but this does not account for systematic errors such as charge normalisation and dead-time correction.

Sample		Layer thickness (TFU)	H (at. %)	Mn (at. %)	O (at. %)	K (at. %)
Pristine MnO_2	Surface	800 ± 100	5.5 ± 0.6	30.5 ± 1.5	61.0 ± 0.3	3.0 ± 0.2
	Bulk	Max. 7750	7.0 ± 0.7	30.0 ± 1.5	60.0 ± 0.3	3.0 ± 0.2
0.9 V, 5 h	Surface	800 ± 100	13.5 ± 1.4	27.8 ± 1.5	55.7 ± 2.8	3.0 ± 0.2
	Bulk	Max. 8000	12.5 ± 1.3	28.2 ± 1.4	56.3 ± 2.8	3.0 ± 0.2
1.6 V, 5 h	Surface	800 ± 100	6.0 ± 0.6	30.3 ± 1.5	60.7 ± 0.3	3.0 ± 0.2
	Bulk	Max. 7750	7.5 ± 0.8	29.8 ± 1.5	59.7 ± 0.3	3.0 ± 0.2

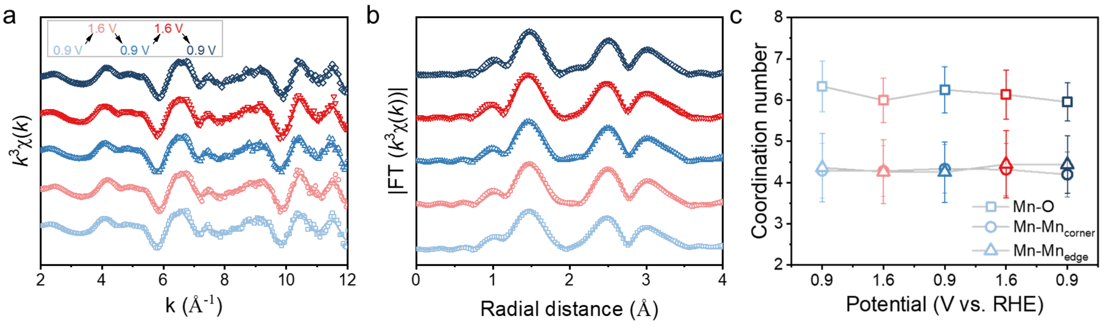

Supplementary Figure 10. Experimental and fitted results of Mn K-edge XAFS. (a) k^3-weighted EXAFS spectra in k space. Dots are experimental data and lines are fitted results. (b) The corresponding Fourier-transformed magnitude in R space. (c) The plot of fitted coordination number results.

Supplementary Table 3. Mn K-edge EXAFS fitted parameters.

Sample	Path	CN	R / Å	σ^2 / Å^2	ΔE_0	R factor / %
	Mn-O	6.3 ± 0.6	1.89 ± 0.01	0.002 ± 0.001		
0.9 V	Mn-Mn (edge)	4.3 ± 0.7	2.89 ± 0.01	0.003 ± 0.001	−7.13	2.08
	Mn-Mn (corner)	4.4 ± 0.8	3.44 ± 0.01	0.003 ± 0.001		
	Mn-O	6.0 ± 0.5	1.89 ± 0.01	0.001 ± 0.001		
1.6 V	Mn-Mn (edge)	4.3 ± 0.6	2.89 ± 0.01	0.002 ± 0.001	−4.15	1.93
	Mn-Mn (corner)	4.3 ± 0.8	3.45 ± 0.01	0.002 ± 0.001		
	Mn-O	6.2 ± 0.5	1.89 ± 0.01	0.002 ± 0.001		
0.9 V	Mn-Mn (edge)	4.3 ± 0.6	2.90 ± 0.01	0.002 ± 0.001	−6.52	1.69
	Mn-Mn (corner)	4.3 ± 0.7	3.45 ± 0.01	0.004 ± 0.001		
	Mn-O	6.1 ± 0.6	1.89 ± 0.01	0.001 ± 0.001		
1.6 V	Mn-Mn (edge)	4.3 ± 0.6	2.90 ± 0.01	0.002 ± 0.001	−7.04	2.12
	Mn-Mn (corner)	4.4 ± 0.8	3.45 ± 0.01	0.002 ± 0.001		
	Mn-O	6.0 ± 0.5	1.89 ± 0.01	0.001 ± 0.001		
0.9 V	Mn-Mn (edge)	4.2 ± 0.5	2.90 ± 0.01	0.002 ± 0.001	−4.88	1.25
	Mn-Mn (corner)	4.4 ± 0.7	3.45 ± 0.01	0.002 ± 0.001		

Mn-O represent the first nearest neighbour coordination shells of O atoms. Mn-Mn (edge) and Mn-Mn (corner) indicate Mn-Mn coordination in edge-shared octahedra and corner-shared octahedra, respectively. CN, coordination number; R, the distance between absorber and backscatter atoms; σ^2, Debye-Waller factor to account for thermal and structural disorders; ΔE_0, inner potential correction; R-factor indicates the goodness of fitting. S_0^2 is fixed to be 0.6. The fitting range for Mn K-edge is $3.0 \leq k \leq 13.0$, $1.0 \leq R \leq 4.0$.

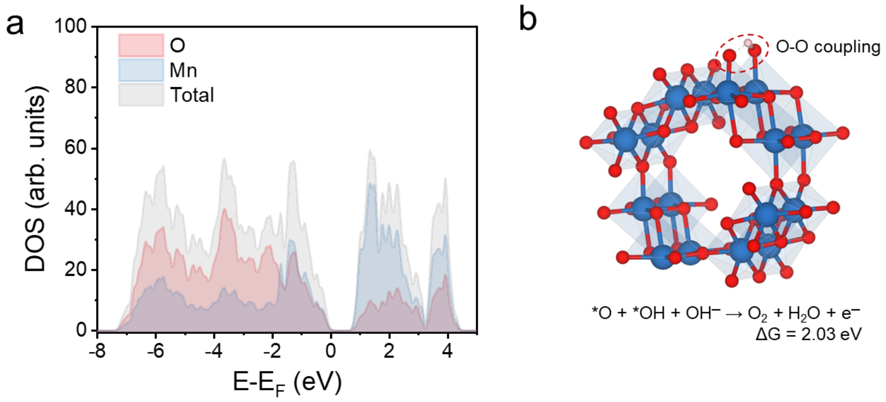

Supplementary Figure 11. The DFT results for excluding the lattice oxygen mechanism. (a) The calculated projected DOS of pristine α-MnO_2. (b) Structure model of direct O-O coupling on MnO_2 surface.

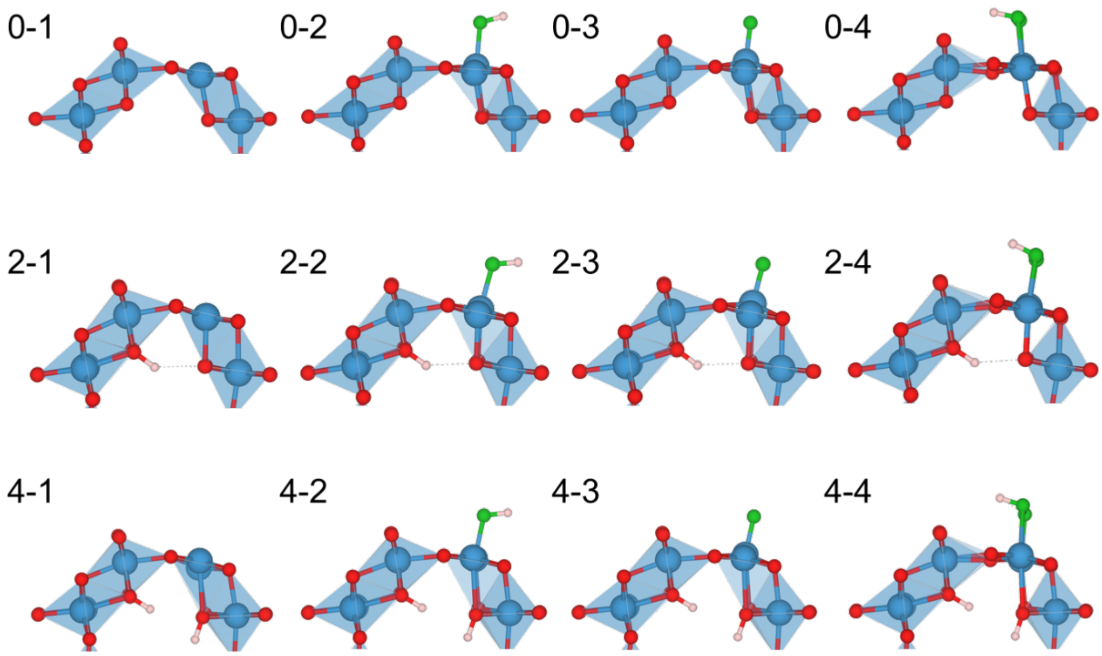

Supplementary Figure 12. The structure models of intermediates in OER pathways on MnO_2 (010) facet with no surface proton. The first digit in labels (0, 2, 4) represents the structures with 0, 2 and 4 protons adsorbed on the di-μ-oxo sites in a tunnel structure, respectively. The second digit in labels (1, 2, 3, 4) represents the pristine (010) facet, *OH, *O and *OOH, respectively.

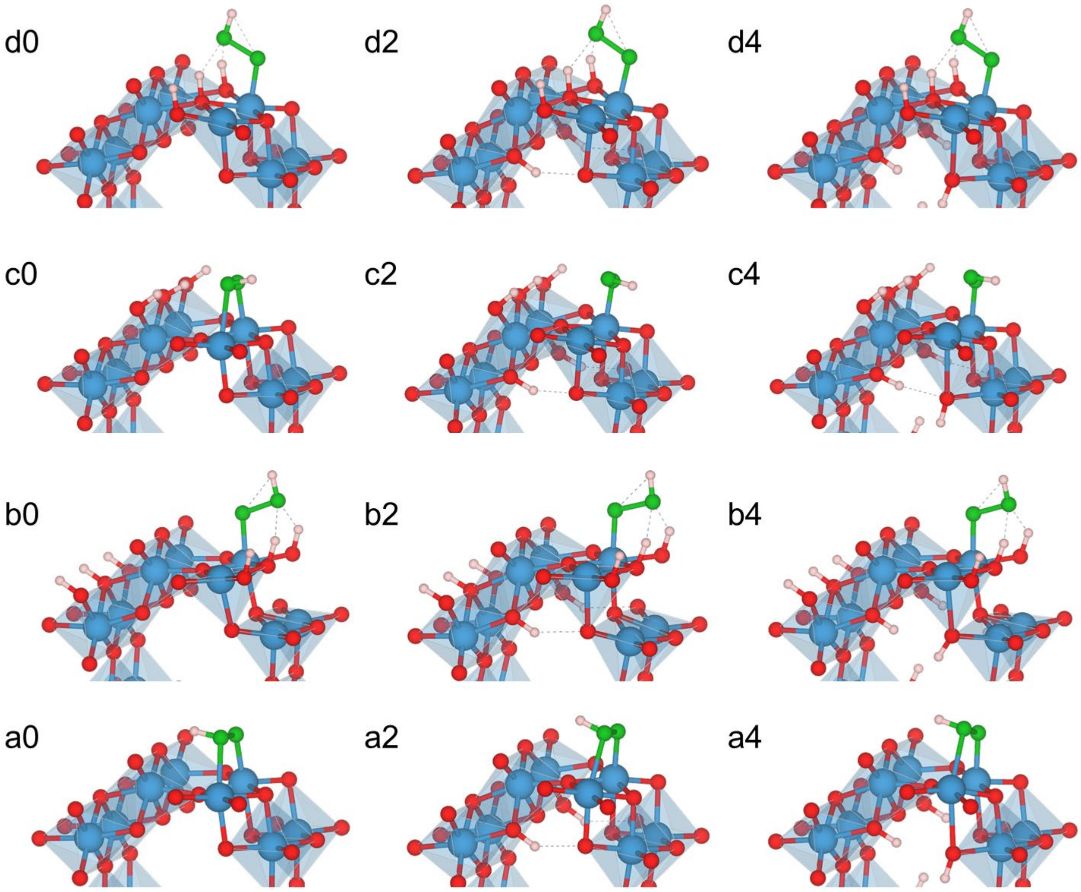

Supplementary Figure 13. The lateral view of *OOH intermediates of different surface and tunnel structures. a, b, c and d illustrate the adsorption states with no surface protons, surface protons on site 1, surface protons on site 2 and surface protons on site 3, respectively. 0, 2, 4 represents structures with 0, 2 and 4 protons adsorbed in a tunnel structure.

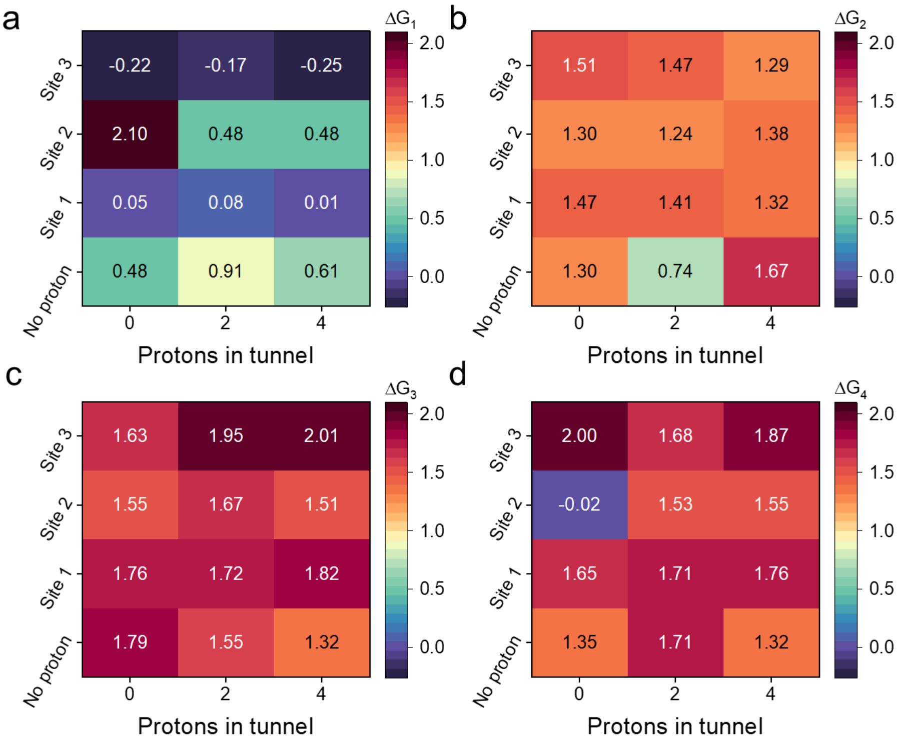

Supplementary Figure 14. The relationship between the free energy of adsorption states on MnO_2 (010) facet and the different protons adsorption states and configuration of surface and tunnel protons. (a-d) show the heat maps of ΔG_1, ΔG_2, ΔG_3, ΔG_4 (the unit is eV), respectively. ΔG_1, ΔG_2, ΔG_3 and ΔG_4 represent the free energy change between four supposed states. The horizontal axis represents the proton number in the tunnel unit, and the vertical axis represents the surface proton configurations.

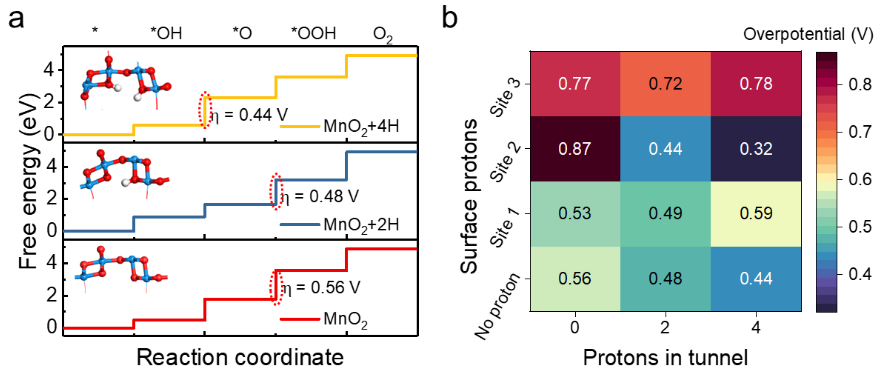

Supplementary Figure 15. The calculated free-energy of OER process of α-MnO_2. (a) The calculated OER free-energy diagram for different α-MnO_2 structures with tunnel-adsorbed protons. (b) The calculated overpotential of α-MnO_2 (010) facet with different proton configurations on the surface and in tunnels.

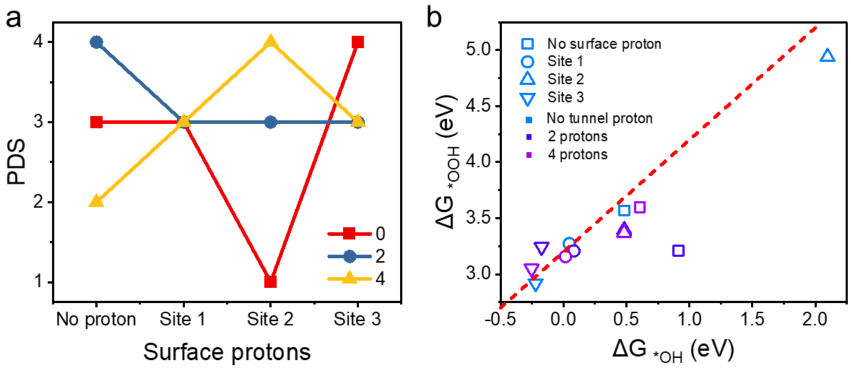

Supplementary Figure 16. The potential-determining step and scaling relationship in OER. (a) The proton configuration dominated potential-determining steps (PDS) and (b) adsorption energy scaling relation between *OH and *OOH.

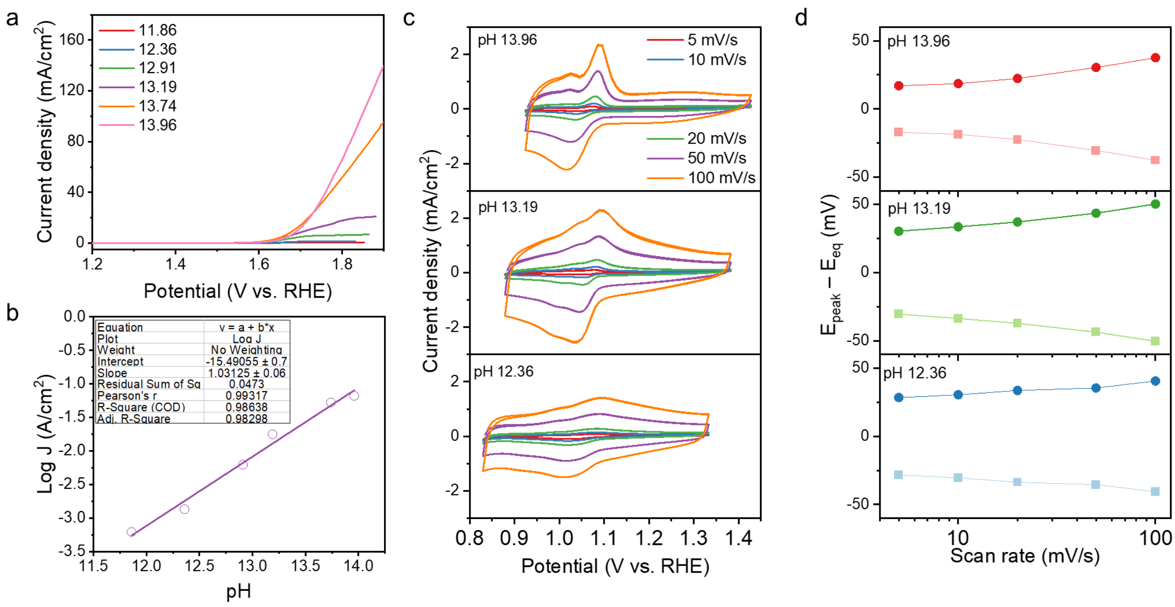

Supplementary Figure 17. pH-dependent OER measurement. (a) pH dependence for CV curves of MnO_2 in KOH solution with different concentrations. The electrolyte was prepared by adding potassium sulphate to maintain the constant K^+ strength. (b) The relationship between current density at 1.8 V vs. RHE and pH. (c) The CV curves of MnO_2 were measured at different pH KOH solutions with different scanning rates. (d) The corresponding peak separation at different scan rates. E_{peak}-E_{eq} represents the difference between peak potential and equilibrium potential.

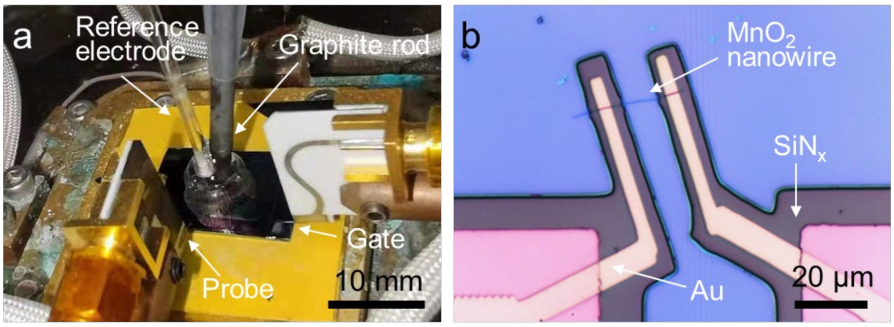

Supplementary Figure 18. Optical image of the single nanowire device. (a) Three-electrode with a gate electrode configuration and (b) the optical micrograph of single MnO_2 nanowire electrode.

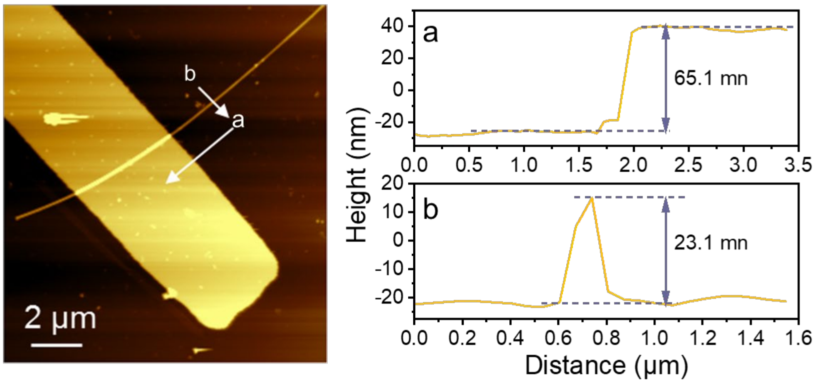

Supplementary Figure 19. AFM image of single MnO_2 nanowire electrode and the corresponding height profile. (a) and (b) are the height profiles corresponding to the arrows a and b in AFM images.

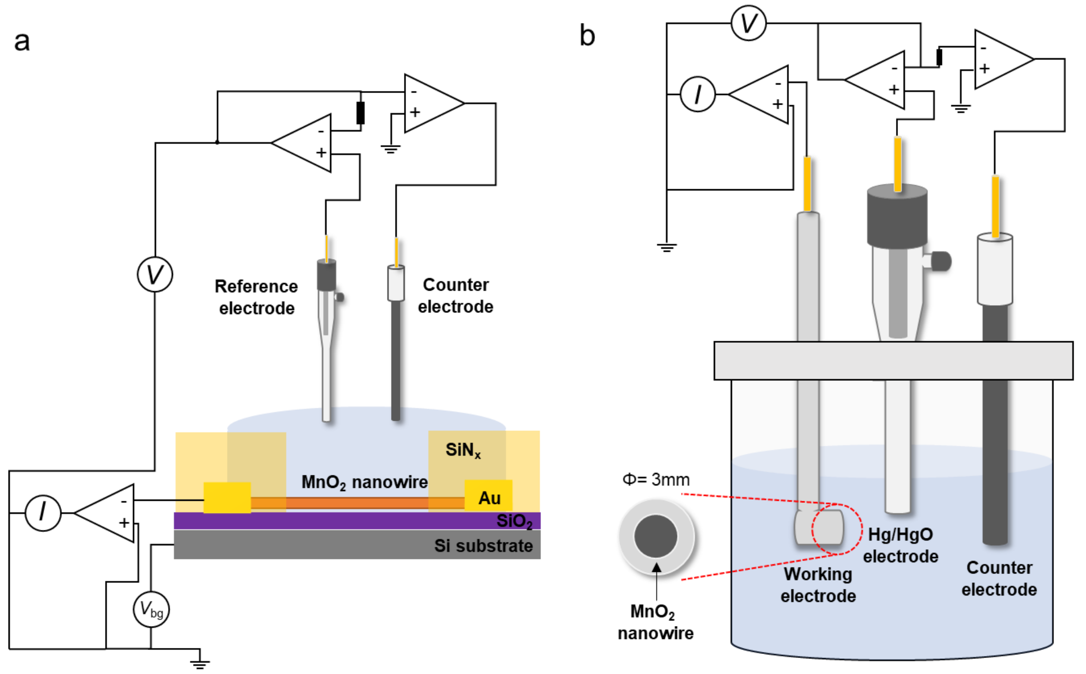

Supplementary Figure 20. The schematic illustration of the single nanowire electrochemical measurement and the traditional three-electrode cell. (a) The on-chip single MnO_2 nanowire device provides a planar electrochemical interface. One single nanowire serviced as the working electrode and a droplet of electrolyte provides electrochemical environment. In this device, the heavily doped Si substrate, and the 300 nm SiO_2 layer acts as the back gate which provides an adjustable electric field. (b) Traditional three-electrode cell is based on a polished glassy carbon electrode (diameter is 3 mm). MnO_2 film was drop-casting on this electrode for electrochemical measurement.

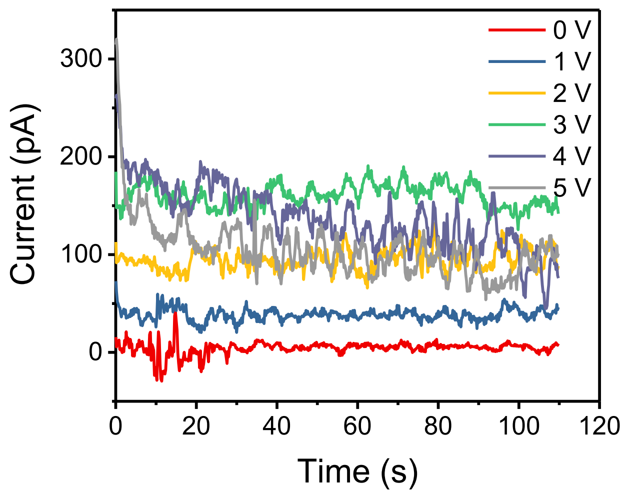

Supplementary Figure 21. Gate current of the single nanowire device at different gate voltage in 1 M KOH electrolyte. The gate current is measured to evaluate the leak current from the gate electrode (Si/SiO_2) during electrochemical measurement. The measured gate current is picoamp level.

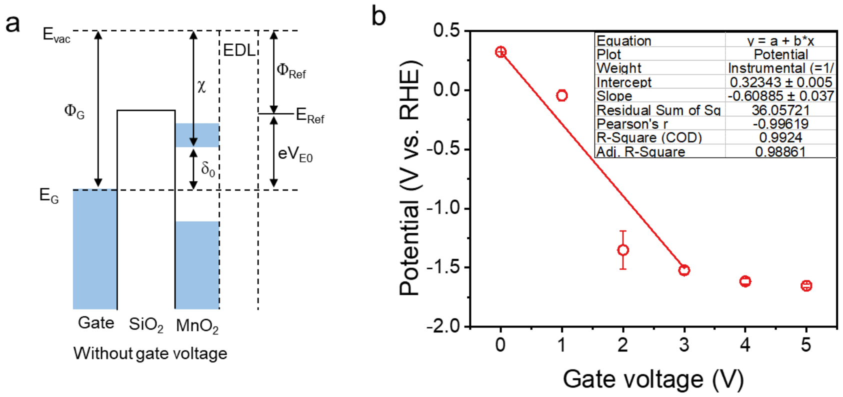

Supplementary Figure 22. The analysis of energy level alignment. (a) Energy diagram of MnO_2 electrochemical system without applied back gate voltage. The symbols in this figure are: vacuum level (E_{vac}), Fermi level of back gate (E_G), work functions of back gate (Φ_G) and Reference electrode (Φ_{ref}), electron affinity of MnO_2 (χ), Fermi level offset ($\delta = E_c - E_F$). Electrical double layer (EDL), electrode potential (V_E), and vacuum level shifts in SiO_2 ($\Delta\phi_G$). (b) The plot of open-circuit potential versus back gate voltage. The fit line shows a linear relationship between open-circuit potential and gate voltage.

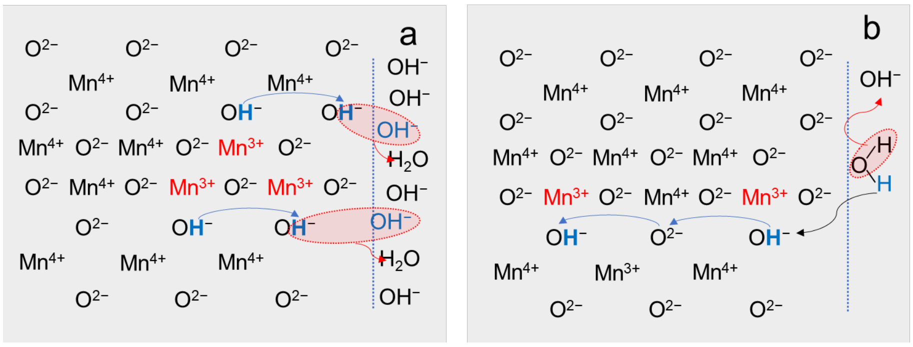

Supplementary Figure 23. The schematic illustration of electron-proton mechanism[31]. (a) In the oxidation process, Mn^{3+} site loses an electron, and the proton will dissociate from oxygen and move to the adjacent oxygen sites. Ultimately, the protons couple with the hydroxyl and generate H_2O molecules. (b) In the reduction process, the protons are supported by the dissociated H_2O molecules at the solid/liquid interface and the protons are adsorbed by the bridged oxygen and introduced into the lattice.

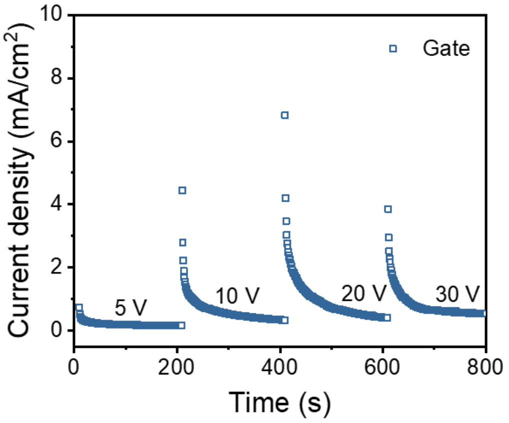

Supplementary Figure 24. The plot of gate current density measured by anion exchange membrane flow cell. The gate current density was measured simultaneously during the staircase scanning gate voltage (5, 10, 20, 30 V).

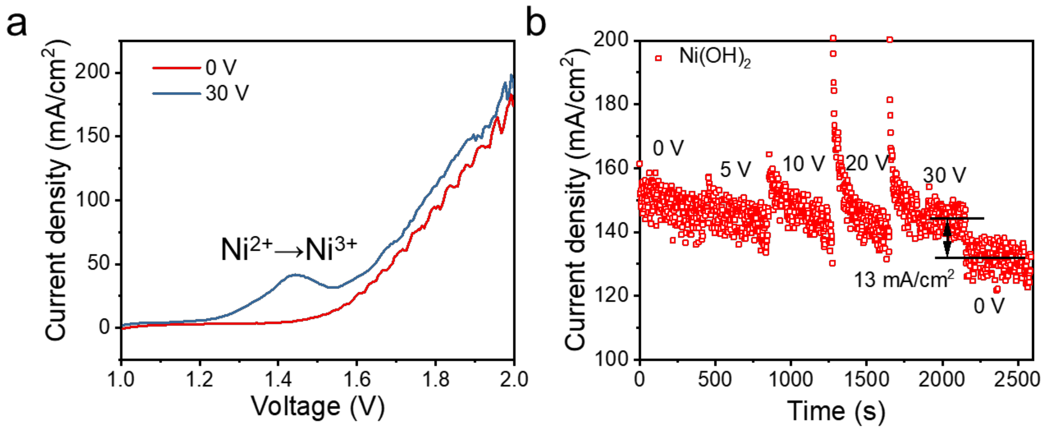

Supplementary Figure 25. The electric-field-enhanced OER of $Ni(OH)_2$. (a) The polarisation curves of overall water splitting under different gate voltages with $Ni(OH)_2$ and commercial Pt/C as anode and cathode, respectively. (b) The plot of chronoamperometry response under different gate voltage with a cell voltage set at a constant value of 2 V. The polarisation curve at 30 V gate voltage shows an enhanced oxidation peak corresponding to Ni^{2+}/Ni^{3+}, as well as an increased current density for overall water splitting.

Crystal structure data (atomic coordinates in Å).

_cell_length_a 5.7146

_cell_length_b 9.6150

_cell_length_c 24.0144

_cell_angle_alpha 90.0000

_cell_angle_beta 90.0000

_cell_angle_gamma 89.9943

Mn	0.25735	0.16882	0.25657
Mn	0.0072	0.66236	0.05114
Mn	0.25712	0.86084	0.11939
Mn	0.00729	0.35142	0.33305
Mn	0.25731	0.34001	0.04341
Mn	0.00764	0.84464	0.24709
Mn	0.25718	0.67412	0.32552
Mn	0.00736	0.1832	0.12712
Mn	0.75735	0.16863	0.25661
Mn	0.5072	0.66242	0.05114
Mn	0.75713	0.8609	0.11939
Mn	0.5073	0.35148	0.33305
Mn	0.75731	0.34007	0.04341
Mn	0.50662	0.8447	0.24709
Mn	0.75718	0.6741	0.32591
Mn	0.50736	0.18326	0.12712
O	0.25741	0.05464	0.12424
O	0.00597	0.55144	0.33924
O	0.25708	0.97057	0.25106
O	0.00724	0.46857	0.04628
O	0.25719	0.67755	0
O	0.00727	0.18426	0.20492
O	0.25731	0.33355	0.37938
O	0.00713	0.84565	0.17053
O	0.00746	0.15937	0.30948
O	0.2572	0.66355	0.10387
O	0.00711	0.85965	0.06666
O	0.25728	0.35926	0.27629
O	0.00736	0.20883	0.04722
O	0.25719	0.7075	0.24902
O	0.00681	0.80546	0.32812
O	0.25729	0.31438	0.12331
O	0.75741	0.0547	0.12424
O	0.50851	0.55151	0.33924
O	0.75708	0.97029	0.25122
O	0.50725	0.46863	0.04628
O	0.75719	0.67761	0
O	0.50743	0.18432	0.20492
O	0.75731	0.3325	0.37934
O	0.50714	0.84571	0.17053
O	0.50728	0.15943	0.30948
O	0.75721	0.66361	0.10387
O	0.50712	0.85971	0.06666
O	0.75728	0.35938	0.27636

O	0.50736	0.20889	0.04722
O	0.75719	0.70731	0.2495
O	0.50744	0.80552	0.32812
O	0.75729	0.31444	0.12331

Supplementary References

1. Chen, D. et al. Probing the charge storage mechanism of a pseudocapacitive MnO_2 electrode using in operando raman spectroscopy. *Chem. Mater.* **27**, 6608-6619 (2015).
2. Ravel, B. & Newville, M. ATHENA, ARTEMIS, HEPHAESTUS: data analysis for X-ray absorption spectroscopy using IFEFFIT. *J. Synchrotron Radiat.* **12**, 537-541 (2005).
3. Anisimov, V. I., Aryasetiawan, F. & Lichtenstein, A. I. First-principles calculations of the electronic structure and spectra of strongly correlated systems: the LDA+U method. *J. Phys.-Condens. Mat.* **9**, 767-808 (1997).
4. Anisimov, V. I., Zaanen, J. & Andersen, O. K. Band theory and Mott insulators: Hubbard U instead of Stoner I. *Phys. Rev. B* **44**, 943-954 (1991).
5. Delley, B. An all-electron numerical method for solving the local density functional for polyatomic molecules. *J. Chem. Phys.* **92**, 508-517 (1990).
6. Delley, B. From molecules to solids with the DMol3 approach. *J. Chem. Phys.* **113**, 7756-7764 (2000).
7. Perdew, J. P., Burke, K. & Ernzerhof, M. Generalized gradient approximation made simple. *Physical Review Letters* **77**, 3865-3868 (1996).
8. Cheng, F. et al. Enhancing electrocatalytic oxygen reduction on MnO_2 with vacancies. *Angew. Chem. Int. Edit.* **125**, 2534-2537 (2013).
9. Grimme, S. Semiempirical GGA-type density functional constructed with a long-range dispersion correction. *J. Comput. Chem.* **27**, 1787-1799 (2006).
10. Monkhorst, H. J. & Pack, J. D. Special points for Brillouin-zone integrations. *Phys. Rev. B* **13**, 5188-5192 (1976).
11. Nørskov, J. K. et al. Origin of the overpotential for oxygen reduction at a fuel-cell cathode. *J. Phys. Chem. B* **108**, 17886-17892 (2004).
12. Weast, R. C. *CRC handbook of chemistry and physics*. (1986).
13. Gao, T. et al. Microstructures and spectroscopic properties of cryptomelane-type manganese dioxide nanofibers. *J. Phys. Chem. C* **112**, 13134-13140 (2008).
14. Suib, S. L. Structure, porosity, and redox in porous manganese oxide octahedral layer and molecular sieve materials. *J. Mater. Chem.* **18**, 1623-1631 (2008).
15. Takashima, T., Hashimoto, K. & Nakamura, R. Mechanisms of pH-dependent activity for water oxidation to molecular oxygen by MnO_2 electrocatalysts. *J. Am. Chem. Soc.* **134**, 1519-1527 (2012).
16. Takashima, T., Hashimoto, K. & Nakamura, R. Inhibition of charge disproportionation of MnO_2 electrocatalysts for efficient water oxidation under neutral conditions. *J. Am. Chem. Soc.* **134**, 18153-18156 (2012).
17. Meng, Y. et al. Structure–property relationship of bifunctional MnO_2 nanostructures: highly efficient, ultra-stable electrochemical water oxidation and oxygen reduction reaction catalysts identified in alkaline media. *J. Am. Chem. Soc.* **136**, 11452-11464 (2014).
18. Yan, G. et al. Phase and morphology transformation of MnO_2 induced by ionic liquids toward efficient water oxidation. *ACS Catal.* **8**, 10137-10147 (2018).
19. Tao, H. B. et al. Identification of surface reactivity descriptor for transition metal oxides in oxygen evolution reaction. *J. Am. Chem. Soc.* **138**, 9978-9985 (2016).
20. Giordano, L. et al. pH dependence of OER activity of oxides: Current and future perspectives. *Catal. Today* **262**, 2-10 (2016).

21. Koper, M. T. M. Theory of multiple proton–electron transfer reactions and its implications for electrocatalysis. *Chem. Sci.* **4**, 2710 (2013).
22. Huynh, M., Bediako, D. K. & Nocera, D. G. A functionally stable manganese oxide oxygen evolution catalyst in acid. *J. Am. Chem. Soc.* **136**, 6002-6010 (2014).
23. Shinagawa, T., Garcia-Esparza, A. T. & Takanabe, K. Insight on Tafel slopes from a microkinetic analysis of aqueous electrocatalysis for energy conversion. *Sci. Rep.* **5**, 13801 (2015).
24. Roche, I., Chaînet, E., Chatenet, M. & Vondrák, J. Carbon-supported manganese oxide nanoparticles as electrocatalysts for the oxygen reduction reaction (ORR) in alkaline medium: physical characterizations and ORR mechanism. *J. Phys. Chem. C* **111**, 1434-1443 (2007).
25. Hong, W. T. et al. Toward the rational design of non-precious transition metal oxides for oxygen electrocatalysis. *Energy Environ. Sci.* **8**, 1404-1427 (2015).
26. Haumann, M. et al. Photosynthetic O_2 formation tracked by time-resolved x-ray experiments. *Science* **310**, 1019-1021 (2005).
27. Zhang, T., Cheng, F., Du, J., Hu, Y. & Chen, J. Efficiently enhancing oxygen reduction electrocatalytic activity of MnO_2 Using facile hydrogenation. *Adv. Energy Mater.* **5**, (2015).
28. Li, Y. F. & Liu, Z. P. Active site revealed for water oxidation on electrochemically induced delta-MnO_2: role of spinel-to-layer phase transition. *J. Am. Chem. Soc.* **140**, 1783-1792 (2018).
29. Balachandran, D., Morgan, D. & Ceder, G. First principles study of H-insertion in MnO_2. *J. Solid State Chem.* **166**, 91-103 (2002).
30. Cockayne, E. & Li, L. First-principles DFT+U studies of the atomic, electronic, and magnetic structure of α-MnO_2 (cryptomelane). *Chem. Phys. Lett.* **544**, 53-58 (2012).
31. Kozawa, A. & Powers, R. The manganese dioxide electrode in alkaline electrolyte; The electron - proton mechanism for the discharge process from MnO_2 to $MnO_{1.5}$. *J. Electrochem. Soc.* **113**, 870-878 (1966).
